# Association between type 2 diabetes mellitus and body composition based on MRI fat fraction mapping

**DOI:** 10.3389/fpubh.2024.1332346

**Published:** 2024-01-23

**Authors:** Qi An, Qin-He Zhang, Yue Wang, Han-Yue Zhang, Yu-Hui Liu, Zi-Ting Zhang, Mei-Ling Zhang, Liang-Jie Lin, Hui He, Yi-Fan Yang, Peng Sun, Zhen-Yu Zhou, Qing-Wei Song, Ai-Lian Liu

**Affiliations:** ^1^Department of Radiology, The First Affiliated Hospital of Dalian Medical University, Dalian, China; ^2^Department of Medical Imaging, Dalian Medical University, Dalian, China; ^3^Department of Thyroid, Metabolic Diseases and Hernia Surgery, The First Affiliated Hospital of Dalian Medical University, Dalian, China; ^4^Philips Healthcare, Beijing, China

**Keywords:** ectopic fat deposition, abdominal muscle, bone marrow adipose tissue, magnetic resonance imaging, imaging biomarker

## Abstract

**Purpose:**

To explore the association between type 2 diabetes mellitus (T2DM) and body composition based on magnetic resonance fat fraction (FF) mapping.

**Methods:**

A total of 341 subjects, who underwent abdominal MRI examination with FF mapping were enrolled in this study, including 68 T2DM patients and 273 non-T2DM patients. The FFs and areas of visceral adipose tissue (VAT), subcutaneous adipose tissue (SAT) and abdominal muscle (AM) were measured at the level of the L1-L2 vertebral. The FF of bone marrow adipose tissue (BMAT) was determined by the averaged FF values measured at the level of T12 and L1 vertebral, respectively. The whole hepatic fat fraction (HFF) and pancreatic fat fraction (PFF) were measured based on 3D semi-automatic segmentation on the FF mapping. All data were analyzed by GraphPad Prism and MedCalc.

**Results:**

VAT area, VAT FF, HFF, PFF of T2DM group were higher than those of non-T2DM group after adjusting for age and sex (*P* < 0.05). However, there was no differences in SAT area, SAT FF, BMAT FF, AM area and AM FF between the two groups (*P* > 0.05). VAT area and PFF were independent risk factors of T2DM (all *P* < 0.05). The area under the curve (AUC) of the receiver operating characteristic (ROC) for VAT area and PFF in differentiating between T2DM and non-T2DM were 0.685 and 0.787, respectively, and the AUC of PFF was higher than VAT area (*P* < 0.05). Additionally, in seemingly healthy individuals, the SAT area, VAT area, and AM area were found to be significantly associated with being overweight and/or obese (BMI ≥ 25) (all *P* < 0.05).

**Conclusions:**

In this study, it was found that there were significant associations between T2DM and VAT area, VAT FF, HFF and PFF. In addition, VAT area and PFF were the independent risk factors of T2DM. Especially, PFF showed a high diagnostic performance in discrimination between T2DM and non-T2DM. These findings may highlight the crucial role of PFF in the pathophysiology of T2DM, and it might be served as a potential imaging biomarker of the prevention and treatment of T2DM. Additionally, in individuals without diabetes, focusing on SAT area, VAT area and AM area may help identify potential health risks and provide a basis for targeted weight management and prevention measures.

## 1 Introduction

The global prevalence of diabetes has continued to increase over the past few decades. According to the International Diabetes Federation, as of 2021, the global prevalence of diabetes has exceeded 10%, of which 90% is type 2 diabetes mellitus (T2DM). It is estimated that by 2045, the prevalence of diabetes will increase to 12.2% and will continue to rise in the future (1, 2). T2DM and its complications have posed a serious threat to global public health.

Previous studies have showed that excessive fat accumulation may increase Insulin resistance (IR), which was considered as the key pathogenesis of T2DM (3–5), consequently promoting the onset and progression of T2DM (6). It was found that the accumulation of visceral adipose tissue (VAT) and ectopic fat deposition, such as liver, pancreas, heart, skeletal muscle, are closely related to IR and T2DM (7, 8). However, there is still controversy surrounding the relationship between ectopic fat deposition and T2DM, particularly in pancreatic fat deposition (9–13). The reasons may be attributed to differences in study population, ethnicity, disease status, and the quantitative techniques employed. Therefore, quantitative assessment of fat accumulation is crucial for the prevention and treatment of T2DM.

In addition to adipose tissue, recently, the relationship of T2DM with muscle and bone, two other important components of body composition, has received increasing attention. Waddell et al. (14) found that skeletal muscle mass of T2DM patients was significantly reduced compared with the non-T2DM group. Additionally, a cross-sectional study in a multi-ethnic population demonstrated that skeletal muscle mass may have an independent role compared to body size or VAT in regulating blood glucose in T2DM (15). Furthermore, Hofbauer et al. (16) emphasized that T2DM may lead to deposition of bone marrow adipose tissue (BMAT), thereby increasing the risk of diabetic fragility fractures.

Although previous studies have highlighted the relationship between body composition and T2DM, most of them were primarily focused on specific components of body composition, such as adipose tissue, muscle or bone, rather than considering them as a holistic concept and evaluating multiple factors of body composition simultaneously (15, 17, 18). It is still unclear which factor serves as the optimal biomarker for identifying T2DM. Therefore, research on comprehensive and quantitative assessment of such body composition factors are of great significance for a deep understanding of the pathogenesis of T2DM and the development of more effective prevention and treatment strategies.

Magnetic resonance imaging (MRI) enables fat fraction (FF) mapping through chemical shift encoding, and the FF is commonly defined as the percentage of proton density of fat molecules relative to the combined proton density of water and fat molecules (19, 20). Compared with traditional imaging techniques such as dual-energy x-ray absorptiometry (DXA) and bioelectrical impedance analysis (BIA), FF mapping by MRI can provide fast and accurate evaluation of the fat composition of the whole body, and it has been widely applied in the assessment of abdominal muscle (AM) (21–24), BMAT (23–25), and ectopic fat deposition (25, 26).

Therefore, the purpose of this study is to use MRI FF mapping to explore the association between T2DM and body composition, including the AM, BMAT content and ectopic adipose deposition, and to identify potential imaging biomarkers for prediction of T2DM.

## 2 Methods

### 2.1 Study design and participants

This single-center, retrospective study collected inpatients who underwent 1.5 or 3.0 T MRI examination of upper abdomen between January 2017 and March 2021, and the scan sequences include MRI FF mapping. Exclusion criteria: 1. lack of clinical data; 2. Age < 18 years; 3. a history of alcoholism (alcohol intake ≥ 210 g/week for men and 140 g/week for women in the past 10 years); 4. cirrhosis, decompensated liver disease, liver malignant tumor, large benign liver tumor, post-hepatectomy and other liver diseases (such as viral hepatitis, drug-induced liver injury, autoimmune liver disease, etc.); 5. history of pancreatic and bile duct diseases (e.g., acute or chronic pancreatitis, autoimmune pancreatitis, pancreatic tumor, pancreatic surgery, pancreatic trauma, biliary and pancreatic duct dilatation, etc.); 6. ascites, abdominal edema, huge abdominal mass, mesenteric surgery, postoperative history of abdominal ostomy, etc.; 7. history of radiotherapy and chemotherapy; 8. weight changes more than 5% within 1 month; 9. vertebral body injury, vertebral body occupation, vertebral body surgery etc.

T2DM was defined as fasting plasma glucose (FPG) ≥ 7.0 mmol/L or being treated with oral hypoglycemic drugs or insulin. Participants who met the diagnostic criteria of T2DM were divided into the T2DM group; otherwise were divided into the non-T2DM group. To further analyze the association between seemingly healthy population and body composition, a stratified analysis was conducted based on BMI, with the non-T2DM group divided into BMI < 25 and BMI ≥ 25 subgroups.

This single-center, retrospective study was approved by the ethics committee of the First Affiliated Hospital of Dalian Medical University, and a waiver of informed consent was remitted.

### 2.2 MRI examinations

Abdominal MRI examinations were performed in supine position with 8-channel phased array coils and abdominal breathing gating (compensation) on a 1.5 or 3.0 T MRI scanner (Signa HDxt, GE Healthcare, Waukesha, WI, USA), or in supine position with 16-channel phased array coils on a 3.0 T MRI scanner (Ingenia CX, Philips Healthcare, Best, the Netherlands). Patients were instructed to fast for 4–6 h, and were trained to exhale and hold their breath before MRI scans. We obtained the MRI fat fraction mapping using IDEAL-IQ sequence and mDixon Quant sequence, with the specific parameters as follows (Table 1): 1.5 T MRI IDEAL-IQ sequence: TR = 13.4 ms, TE = 4.8 ms, FOV = 36 × 36 cm^2^, matrix = 256 × 160, NEX = 1, slice thickness = 10 mm, flip angle = 5°. 3.0 T MRI IDEAL-IQ sequence: TR = 6.9 ms, TE = 3 ms, FOV = 36 × 36 cm^2^, matrix = 256 × 160, NEX = 1, slice thickness = 10 mm, flip angle = 3°. 3.0 T MRI mDixon Quant sequence: TR = 6 ms, TE = 1.05 ms, FOV = 37 × 30 cm^2^, matrix = 176 × 130, NEX = 1, slice thickness = 5 mm, flip angle = 3°. Multiple acquired echoe signals were collected during a single breath-hold, and the water-phase, fat-phase, in-phase, out-phase, R2^*^ and fat fraction mapping were generated after reconstruction.

**Table 1:** MRI fat fraction mapping scan parameters.

**MR sequences**	**TR (ms)**	**TE (ms)**	**FOV (cm^2^)**	**Matrix**	**NEX**	**Slice thickness (mm)**	**flip angle (°)**
IDEAL-IQ (1.5 T)	13.4	4.8	36 × 36	256 × 160	1	10	5
IDEAL-IQ (3.0 T)	6.9	3.0	36 × 36	256 × 160	1	10	3
mDixon quant (3.0 T)	6.0	1.05	37 × 30	176 × 130	1	5	3

TR, repetition time; TE, echo time; FOV, field of view; NEX, number of excitation.

### 2.3 Data measurements

#### 2.3.1 Visceral adipose tissue, subcutaneous adipose tissue, hepatic fat fraction, and pancreatic fat fraction measurement

VAT and subcutaneous adipose tissue (SAT) were semi-automatically measured by Image J (National Institutes of Health, USA) (https://imagej.nih.gov/ij), and hepatic fat fraction (HFF) and pancreatic fat fraction (PFF) were semi-automatically measured based on the 3D semi-automatic segmentation using the multimodality tumor tracking software on the Philips post-processing workstation (Intellispace Portal, ISP v9.0), and the VAT area, SAT area, VAT FF, SAT FF, HFF and PFF were automatically calculated according to previous studies (20, 27).

#### 2.3.2 Abdominal muscle measurement

**Figure 1:**
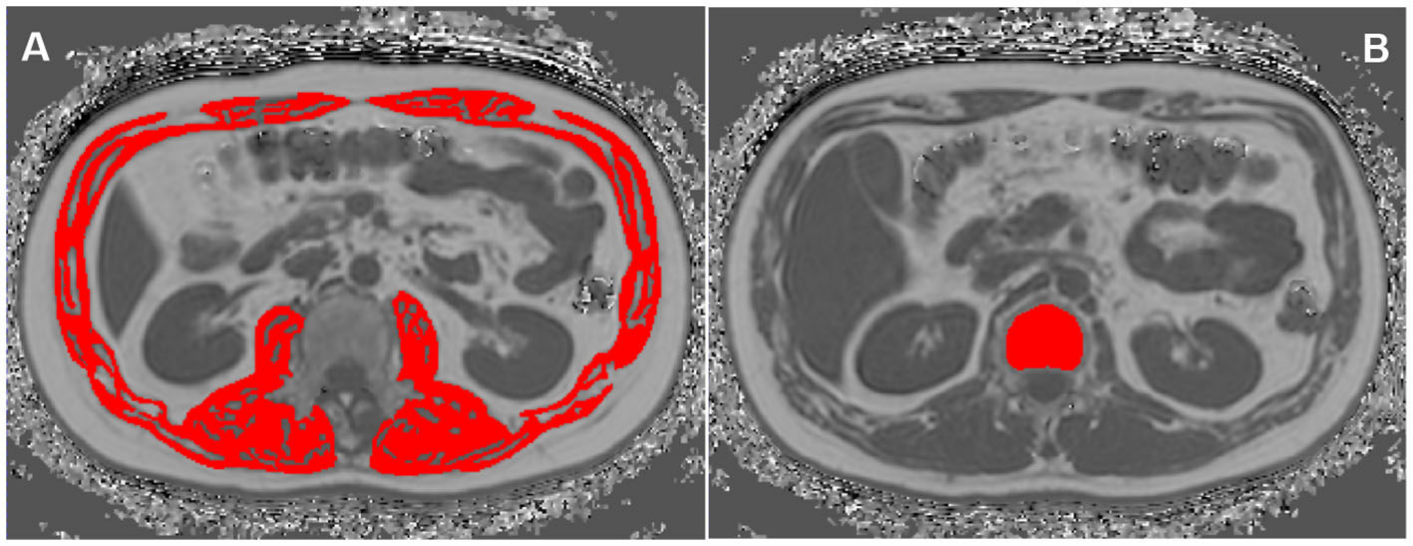
Region of interests (ROIs) of abdominal muscle (AM) **(A)** and vertebral bone marrow adipose tissue (BMAT) **(B)** on the MRI fat fraction (FF) mapping.

AM was manually delineated on MRI axial fat fraction maps at L1-L2 level by using Image J (19, 28, 29), including bilateral erector spinae muscles, quadratus lumborum, psoas major, internal and external oblique muscles, transverse abdominis and rectus abdominis, and then the area and FF for all these muscles were automatically calculated (Figure 1A).

#### 2.3.3 Vertebral bone marrow adipose tissue measurement

The BMAT FF was measured at T12 and L1 vertebral bodies on the Philips post-processing workstation (Intellispace Portal, ISP V9.0) (30). On the axial fat fraction mapping, the region of interests (ROIs) were placed in the center of T12 and L1 vertebral bodies, respectively. And ROIs were drawn along the inner edge of the boundary of the vertebral bodies to contain as many vertebral body area as possible, while avoiding confounding structures such as cortical bone, proliferative osteophyte and other tissues outside the vertebral body. The T12 and L1 spine FF were automatically measured, and the mean spine FF was calculated (Figure 1B).

#### 2.3.4 Other data measurements

All participants were required to fast for ≥12 h before blood drawing and collect blood samples in the morning. FPG, triglyceride (TG), total cholesterol (TC), high density lipoprotein cholesterol (HDL-C) and low density lipoprotein cholesterol (LDL-C) were measured by the laboratory staff in our hospital using standard laboratory procedures. The height, weight, systolic blood pressure (SBP), and diastolic blood pressure (DBP) of all subjects were measured by professionally trained nurses in accordance with international standards. Height was measured using a stadiometer with participants removing their cap and shoes, standing upright in the center of the platform, body relaxed, arms naturally drooping down. The measurement accuracy was ±0.1 cm, and two consecutive measurements were taken and averaged. Weight was measured using an electronic scale with participants removing their cap and shoes, wearing light clothing. The measurement accuracy was ±0.1 kg, and two consecutive measurements were taken and averaged. Body mass index (BMI) was calculated using the formula BMI = weight (kg)/height^2^ (m^2^). SBP and DBP in sitting position of the left upper arm were measured using a calibrated mercury sphygmomanometer. Participants were required to maintain a seated position for at least 5 min before measurement. Two consecutive measurements were taken with a 1–2 min interval, and the average was calculated. The clinical information, including gender, age, smoking status and current alcohol use were acquired from the patient's electronic medical records.

### 2.4 Inter- and intra-observer variability

The intra- and inter-observer variability of the MRI-acquired fat measurements was determined by repeated analysis of 30 randomly selected patients more than 4 weeks apart by the same observer and by the MRI-acquired fat measurements of the same patient by a second independent observer. Two radiologists were blinded to the grouping information.

### 2.5 Statistical analysis

All data were analyzed by GraphPad Prism (Version 8.4.0, GraphPad software, LLC) and MedCalc (Version 20.022, MedCalc Software bvba, Ostend, Belgium). The intraclass correlation coefficient (ICC) was used to assess the consistency of measured data. The Kolmogorow-Smironov test was used to analyze the normality of continuous variables.

Normally distributed data were represented by mean ± standard deviation, and non-normally distributed data were represented by median (25th quantile value, 75th quantile value). Categorical variables were expressed as the number of cases and percentage.

Comparisons between T2DM and non-T2DM groups were determined using the two-sided independent sample *t*-test or the non-parametric Mann–Whitney *U*-test for normally or non-normally distributed continuous variables, and the chi-square test for categorical variables.

To assess the correlations between various body compositions, the adjustment coefficient (*r*) among ectopic fat deposition, AM and BMAT parameters after correction for age, sex and BMI were computed. Correlation coefficients were interpreted as follows: weak, 0–0.4; moderate, 0.4–0.7; strong, 0.7–1.0.

The associations between the body compositions and T2DM were assessed by logistic regression analysis. Receiver operating characteristics (ROC) analysis was performed to calculate the area under the ROC curve (AUC) for body compositions to identify T2DM patients. Additionally, the cut-off value, sensitivity and specificity were also estimated using Youden index. Delong test was used to compare the AUC values.

A two tailed *P* < 0.05 were considered statistically significant.

## 3 Results

### 3.1 Study subjects characteristics

A total of 341 participants were finally enrolled in this study, including 68 patients in the T2DM group (40 men and 28 women) and 273 patients in the non-T2DM group (117 men and 156 women). The average age and BMI of patients in T2DM group were significantly higher than those in non-T2DM group (*P* < 0.05). There were more male patients in the T2DM group (58.8 vs. 42.9% in the non-T2DM group) (*P* < 0.05). The detailed clinical characteristics were shown in Table 2.

**Table 2:** Characteristics of the study subjects.

**Variables**	**T2DM (*n* = 68)**	**Non-T2DM (*n* = 273)**	***P*-value**	*P*-value^*^
**Clinical characteristics**				
Age, years	63.81 ± 12.35	57 (49, 64)	**< 0.001**	–
Sex, *n* (%)			**0.018**	–
Male	40 (58.80)	117 (42.90)	–	–
female	28 (41.20)	156 (57.10)	–	–
BMI, kg/m^2^	25.49 ± 2.54	24.41 ± 3.09	**0.008**	**0.012**
SBP, mmHg	137.10 ± 19.54	120 (113, 130)	**< 0.001**	**< 0.001**
DBP, mmHg	80 (70, 90)	80 (70, 80)	**0.014**	0.067
FPG, mmol/L	7.48 (6.42, 9.30)	4.99 (4.65, 5.42)	**< 0.001**	**< 0.001**
TG, mmol/L	1.63 (1.18, 2.49)	1.12 (0.84, 1.58)	**< 0.001**	**< 0.001**
TC, mmol/L	4.87 (4.32, 5.76)	4.91 ± 1.14	0.399	**0.035**
HDL-C, mmol/L	1.14 ± 0.38	1.31 (1.02, 1.47)	**0.002**	**0.005**
LDL-C, mmol/L	2.80 (2.35, 3.43)	2.70 ± 0.83	0.064	**0.006**
Current smoking status, n (%)	6 (8.80)	28 (10.30)	0.724	0.531
Current alcohol use, *n* (%)	2 (2.90)	13 (4.80)	0.745	0.527
Diabetes treatment, *n* (%)	45 (66.20)	–	–	–
Postmenopausal status, *n* (%)	25 (89.30)	118 (76.60)	0.133	0.445
**Body composition parameters**				
SAT area, cm^2^	118.19 (91.81, 167.93)	123.17 (96.02, 158.43)	0.836	0.172
SAT FF, %	79.77 ± 4.94	82.21 (79.13, 84.66)	**0.007**	0.910
VAT area, cm^2^	187.89 ± 74.01	139.95 ± 66.94	**< 0.001**	**0.001**
VAT FF, %	78.99 (75.85, 80.89)	76.66 (73.07, 79.94)	**0.011**	**0.024**
HFF, %	4.12 (2.92, 7.15)	3.40 (2.60, 5.50)	**0.029**	**0.012**
PFF, %	13.05 (9.80, 19.15)	6.70 (4.20, 9.80)	**< 0.001**	**< 0.001**
AM area, cm^2^	119.49 ± 28.55	104.93 (87.69, 130.59)	**0.016**	0.070
AM FF, %	28.85 (22.86, 33.89)	25.32 (19.78, 32.23)	**0.033**	0.080
BMAT FF, %	46.56 ± 10.51	42.91 ± 11.83	**0.021**	0.429

BMI, body mass index; SBP, systolic blood pressure; DBP, diastolic blood pressure; FPG, fasting plasma glucose; TG, triglycerides; TC, total cholesterol; HDL-C, high-density lipoprotein cholesterol; LDL-C, low-density lipoprotein cholesterol; SAT, subcutaneous adipose tissue; FF, fat fraction; VAT, visceral adipose tissue; HFF, hepatic fat fraction; PFF, pancreatic fat fraction; AM, abdominal muscle; BMAT, bone marrow adipose tissue.

Data were expressed as mean ± SD, median (25th and 75th percentiles) or *n* (%); *P*-value shows comparison of the T2DM and non-T2DM groups.

^*^Adjusted for age and sex. The bold values indicates statistically significant.

### 3.2 Consistency analysis

The data consistency was shown in Table 3. The ICC values were all higher than 0.75, which suggested good inter-observer and intra-observer agreement.

**Table 3:** Two-observer measurement consistency.

**Body composition parameters**	**Radiologist A1**	**Radiologist A2**	ICC 1^*^	**Radiologist B**	ICC 2^*^
SAT area, cm^2^	131.28 (109.95, 149.86)	128.54 (107.38, 154.27)	0.996	130.72 (110.23, 153.03)	0.993
SAT FF, %	82.16 (80.52, 84.14)	83.02 (79.14, 84.85)	0.912	83.45 (79.52, 84.59)	0.902
VAT area, cm^2^	137.26 ± 75.46	139.30 ± 74.58	0.997	139.43 ± 75.77	0.998
VAT FF, %	78.18 (73.85, 80.96)	77.36 (72.55, 80.24)	0.756	77.28 (72.76, 80.07)	0.832
HFF, %	3.50 (2.55, 5.55)	3.80 (2.85, 5.55)	0.987	3.50 (2.75, 5.55)	0.991
PFF, %	6.30 (4.05,11.20)	6.40 (4.25, 10.70)	0.992	6.30 (4.20, 11.75)	0.996
AM area, cm^2^	116.14 ± 28.87	115.54 ± 26.83	0.966	115.39 ± 27.12	0.972
AM FF, %	25.96 (19.79, 30.84)	23.66 (18.44, 29.73)	0.900	24.32 (19.05, 29.28)	0.918
BMAT FF, %	46.32 (39.56, 50.31)	46.29 (39.86, 50.42)	0.987	43.85 ± 10.37	0.928

^*^ICC 1 shows ICC value of Intra-observer and ICC 2 shows ICC value of inter-observer.

### 3.3 Correlations among ectopic fat deposition, AM and BMAT parameters

**Figure 2:**
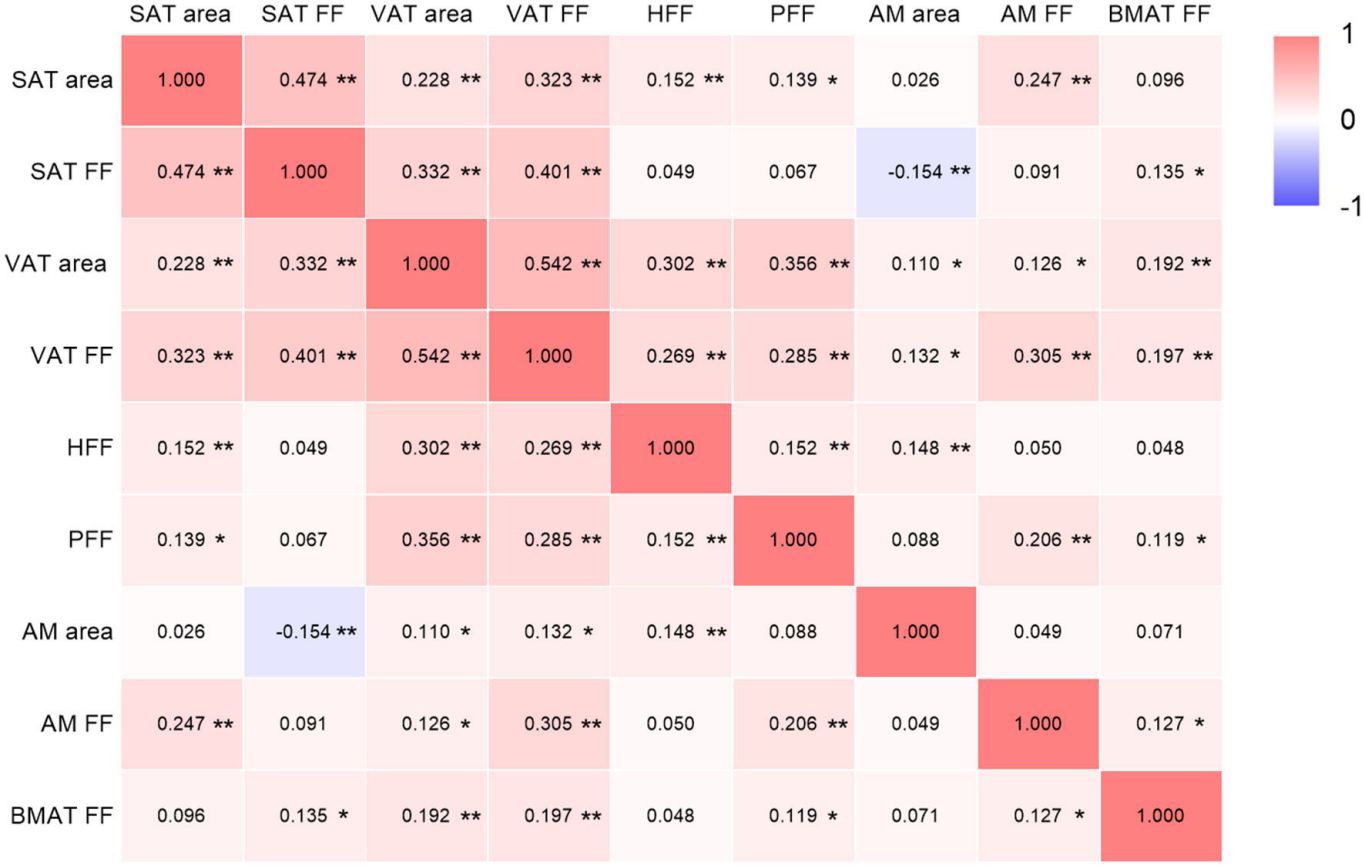
Correlations among ectopic fat deposition, AM and BMAT parameters (adjusted for age, sex and BMI). SAT, subcutaneous adipose tissue; FF, fat fraction; VAT, visceral adipose tissue; HFF, hepatic fat fraction; PFF, pancreatic fat fraction; AM, abdominal muscle; BMAT, bone marrow adipose tissue. The color depth of each cell indicates that the correlation coefficients from low (*r* = −1; purple) to high (*r* = 1; red); *P < 0.05; **P < 0.01.

SAT area, SAT FF, VAT area, VAT FF, HFF, PFF, AM area, AM FF and BMAT FF were correlated after adjusting for age, sex and BMI (*P* < 0.05), but patterns of these correlations were different. It was found that both VAT area and FF were correlated with other quantitative parameters (*P* < 0.05) (Figure 2).

### 3.4 Comparison of body composition parameters between the T2DM group and non-T2DM group

**Figure 3:**
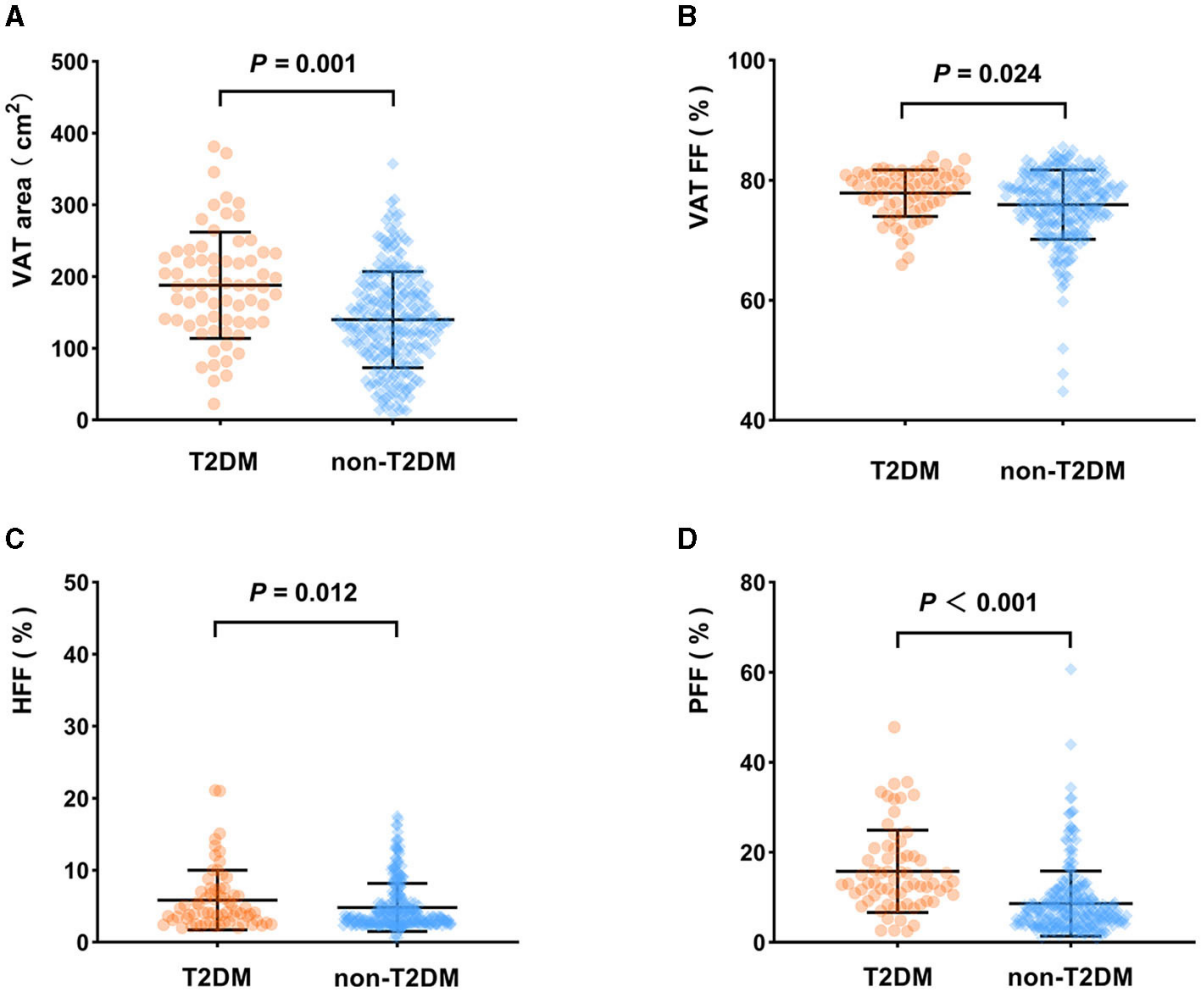
Comparison of body composition parameters between the T2DM group and non-T2DM group (adjusted for age and sex). VAT area **(A)**, VAT FF **(B)**, HFF **(C)**, PFF **(D)** of the T2DM group were higher than those of the non-T2DM group (*P* < 0.05).

VAT area, VAT FF, HFF, PFF, BMAT FF, AM area and AM FF of the T2DM group were 187.89 cm^2^, 78.99%, 4.12%, 13.05%, 46.56%, 119.49 cm^2^, 28.85%, respectively, which were higher than those of the non-T2DM group (139.95 m^2^, 76.66%, 3.40%, 6.70%, 42.91%, 104.93 cm^2^, and 25.32%, respectively), but SAT FF was lower in the T2DM group than in the non-T2DM group (79.77 vs. 82.21%, *P* < 0.05). However, after adjusting for age and gender, the differences between the two groups in SAT FF, AM area, AM FF, and BMAT FF were no longer significant (*P* > 0.05). Additionally, there was no significant difference in SAT area between the two groups, regardless of whether age and gender were adjusted for (*P* > 0.05) (Table 2; Figure 3).

### 3.5 Association between T2DM and body compositions

Multivariate analysis showed that VAT area (OR: 1.005, 95% CI: 1.001–1.010) and PFF (OR: 1.062, 95% CI: 1.025–1.100) were independently associated with T2DM after adjusting for the confounding factors of age, sex, BMI, VAT FF, HFF (Table 4).

**Table 4:** Association between T2DM and body compositions (adjusted for age, sex, and BMI).

**Variables**	**Multivariate analysis**	
	**OR (95% CI)**	***P*** **-value**
VAT area	1.005 (1.001–1.010)	**0.020**
VAT FF	0.972 (0.899–1.051)	0.478
HFF	1.051 (0.966–1.143)	0.248
PFF	1.062 (1.025–1.100)	**0.001**

VAT, visceral adipose tissue; FF, fat fraction; HFF, hepatic fat fraction; PFF, pancreatic fat fraction; OR, odds ratio; CI, confidence interval. The bold values indicates statistically significant.

**Figure 4:**
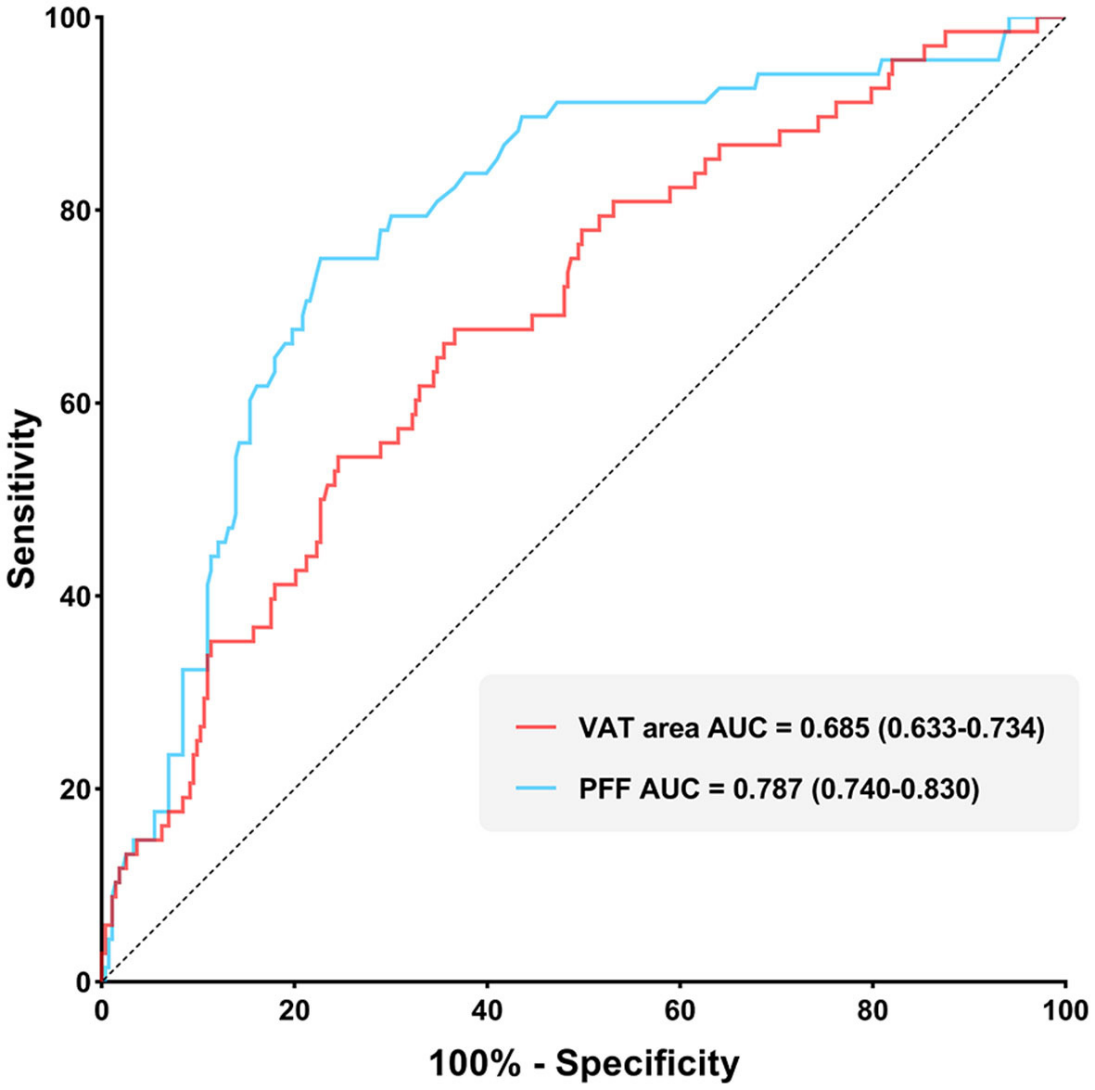
Receiver operating characteristic (ROC) curves of VAT area and PFF for predicting T2DM.

It was found that the AUC of VAT area for identifying T2DM was 0.685 (0.633–0.734) with the sensitivity and specificity of 67.65 and 63.37%, respectively, when using the cut-off value of 159.18 cm^2^. The AUC of PFF for identifying T2DM was 0.787 (0.740–0.830) with the sensitivity and specificity of 75.00 and 77.29%, respectively, when using the cut off value of 10.10% (Table 5; Figure 4).

**Table 5:** The efficacy analysis of VAT area and PFF for predicting T2DM.

**Parameters**	**AUC (95%CI)**	**Cut-off value**	**Sensitivity (%)**	**Specificity (%)**	***P*-value**
VAT area, cm^2^	0.685 (0.633–0.734)	159.18	67.65	63.37	< 0.001
PFF, %	0.787 (0.740–0.830)	10.10	75.00	77.29	< 0.001

AUC, area under the curve; CI, confidence interval; VAT, visceral adipose tissue; FF, fat fraction; PFF, pancreatic fat fraction.

**Figure 5:**
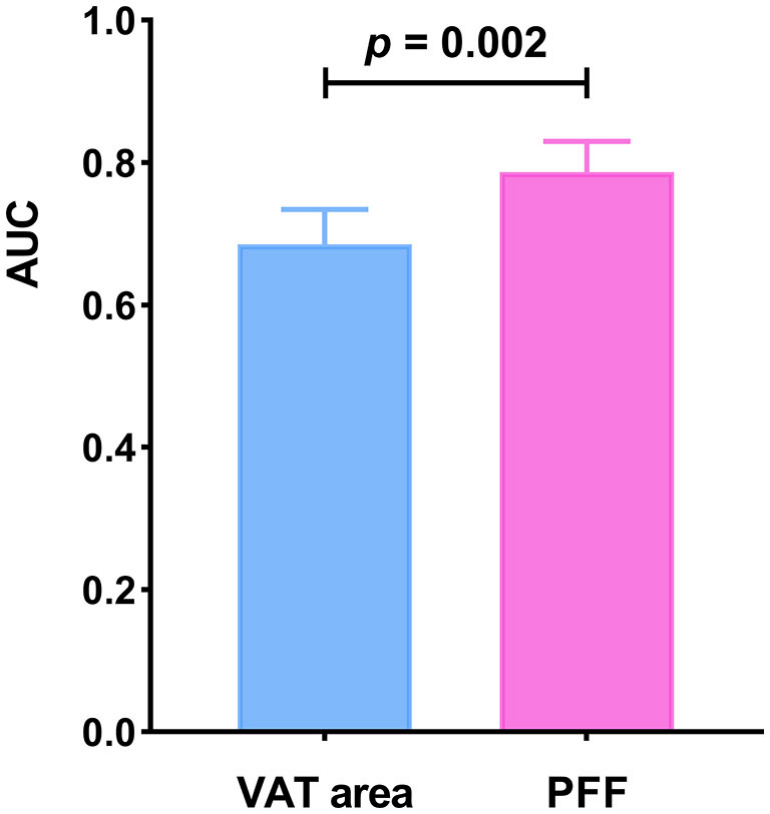
Comparison of diagnostic efficacy among VAT area and PFF for prediction of T2DM (*P* < 0.05).

Furthermore, Delong test was used to compare the diagnostic performance of VAT area and PFF for prediction of T2DM. It demonstrated that PFF has significantly higher diagnostic efficacy for T2DM than VAT area (*P* < 0.05) (Figure 5).

### 3.6 Relationship between non-T2DM and body compositions

**Figure 6:**
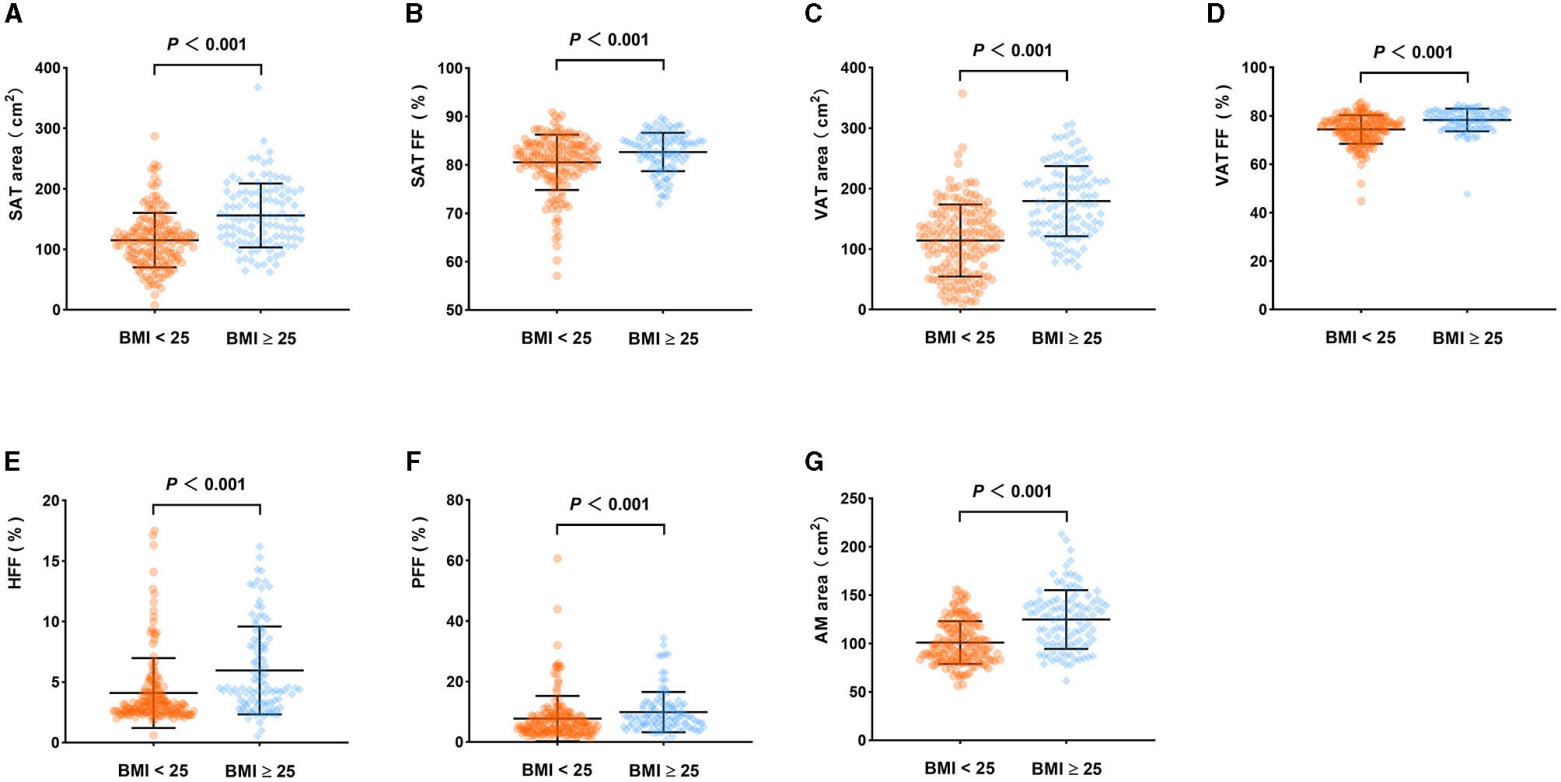
Comparison of body composition parameters between the BMI < 25 group and BMI ≥ 25 group (adjusted for sex). SAT area **(A)**, SAT FF **(B)**, VAT area **(C)**, VAT FF **(D)**, HFF **(E)**, PFF **(F)**, AMAT area **(G)** of the BMI ≥ 25 group were higher than those of the BMI < 25 group (*P* < 0.05).

There was no statistically significant difference in age between the BMI < 25 and BMI ≥ 25 groups (*P* > 0.05), while the difference in gender between the two groups was statistically significant (*P* < 0.05). Moreover, the SAT area, SAT FF, VAT area, VAT FF, HFF, PFF, and AMAT area of the BMI ≥ 25 group were significantly higher than those of BMI < 25 group (*P* < 0.05). However, there was no statistically significant differences in AM FF and BMAT FF between the two groups (*P* > 0.05). It is noteworthy that this relationship remains unchanged even after adjusting for sex (Table 6; Figure 6).

**Table 6:** Characteristics of the non-T2DM subjects.

**Variables**	**All subjects (*n* = 273)**	**BMI < 25 (*n* = 165)**	**BMI ≥25 (*n* = 108)**	***P*-value**	*P*-value^*^
**Clinical characteristics**					
Age, years	57 (49, 64)	57 (49, 64)	55.95 ± 12.47	0.888	
Sex, *n* (%)				**0.029**	
Male	117 (42.86)	62 (37.58)	55 (50.93)	–	
Female	156 (57.14)	103 (62.42)	53 (49.07)	–	
BMI, kg/m^2^	24.41 ± 3.09	22.76 (21.09, 23.84)	26.83 (26.12, 28.25)	**< 0.001**	0.957
SBP, mmHg	120 (113, 130)	120 (110, 130)	130 (120, 140)	**< 0.001**	**0.014**
DBP, mmHg	80 (70, 80)	80 (70, 80)	80 (72, 87.5)	**0.001**	**0.006**
FPG, mmol/L	5.06 ± 0.63	4.92 ± 0.51	5.28 ± 0.72	**< 0.001**	**< 0.001**
TG, mmol/L	1.12 (0.84, 1.58)	1.01 (0.77, 1.47)	1.31 (0.93, 1.88)	**< 0.001**	**0.004**
TC, mmol/L	4.91 ± 1.14	4.72 (4.13, 5.59)	4.94 ± 1.18	0.512	0.429
HDL-C, mmol/L	1.31 (1.02, 1.47)	1.36 (1.04, 1.47)	1.26 ± 0.39	0.179	0.617
LDL-C, mmol/L	2.70 ± 0.83	2.62 (2.16, 3.11)	2.71 ± 0.82	0.491	0.657
Current smoking status, *n* (%)	28 (10.20)	20 (12.10)	8 (7.40)	0.209	**0.034**
Current alcohol use, *n* (%)	13 (4.70)	8 (4.80)	5 (4.60)	0.934	0.566
Postmenopausal status, *n* (%)	118 (43.20)	74 (73.30)	44 (83.00)	0.174	0.178
**Body composition parameters**					
SAT area, cm^2^	123.17 (96.02, 158.43)	114.47 (84.13, 133.52)	155.86 ± 52.77	**< 0.001**	**< 0.001**
SAT FF, %	82.21 (79.13, 84.66)	81.63 (78.38, 84.20)	83.00 (80.38, 85.52)	**0.001**	**< 0.001**
VAT area, cm^2^	139.95 ± 66.94	114.19 ± 59.54	179.30 ± 58.08	**< 0.001**	**< 0.001**
VAT FF, %	76.66 (73.07, 79.94)	75.45 (70.89, 78.16)	79.61 (75.00, 81.65)	**< 0.001**	**< 0.001**
HFF, %	3.40 (2.60, 5.50)	3.10 (2.50, 4.20)	4.45 (3.30, 8.20)	**< 0.001**	**< 0.001**
PFF, %	6.70 (4.20, 9.80)	5.80 (3.50, 8.80)	8.25 (5.65, 12.15)	**< 0.001**	**< 0.001**
AM area, cm^2^	104.93 (87.69, 130.59)	96.93 (83.57, 117.87)	124.77 ± 30.33	**< 0.001**	**< 0.001**
AM FF, %	25.32 (19.78, 32.23)	24.30 (18.79, 32.23)	26.05 (21.35, 32.18)	0.161	0.058
BMAT FF, %	42.91 ± 11.83	42.66 ± 12.90	43.31 ± 10.01	0.639	0.618

BMI, body mass index; SBP, systolic blood pressure; DBP, diastolic blood pressure; FPG, fasting plasma glucose; TG, triglycerides; TC, total cholesterol; HDL-C, high-density lipoprotein cholesterol; LDL-C, low-density lipoprotein cholesterol; SAT, subcutaneous adipose tissue; FF, fat fraction; VAT, visceral adipose tissue; HFF, hepatic fat fraction; PFF, pancreatic fat fraction; AM, abdominal muscle; BMAT, bone marrow adipose tissue.

Data were expressed as mean ± SD, median (25th and 75th percentiles) or n (%); *P*-value shows comparison of the BMI < 25 and BMI ≥ 25 groups.

^*^Adjusted for sex. The bold values indicates statistically significant.

Multivariate analysis showed that SAT area (OR: 1.016, 95% CI: 1.005–1.026), VAT area (OR: 1.016, 95% CI: 1.008–1.024) and AM area (OR: 1.047, 95% CI: 1.026–1.069) were independently associated with BMI ≥ 25 after adjusting for the confounding factors of sex, SAT FF, VAT FF, HFF, PFF (Table 7).

**Table 7:** The correlation between non-T2DM and body compositions (adjusted for sex).

**Variables**	**Multivariate analysis**	
	**OR (95% CI)**	***P*** **-value**
SAT area	1.016 (1.005–1.026)	**0.003**
SAT FF	1.027 (0.926–1.138)	0.617
VAT area	1.016 (1.008–1.024)	**< 0.001**
VAT FF	0.948 (0.875–1.027)	0.191
HFF	1.015 (0.916–1.125)	0.773
PFF	0.966 (0.919–1.017)	0.188
AM area	1.047 (1.026–1.069)	**< 0.001**

SAT, subcutaneous adipose tissue; FF, fat fraction; VAT, visceral adipose tissue; HFF, hepatic fat fraction; PFF, pancreatic fat fraction; AM, abdominal muscle; OR, odds ratio; CI, confidence interval. The bold values indicates statistically significant.

## 4 Discussion

In this study, we found that there were significant associations of T2DM with VAT area, VAT FF, HFF and PFF. In addition, VAT area and PFF were independent risk factors of T2DM, with PFF showing the highest efficacy in prediction of T2DM. Additionally, in seemingly healthy individuals, the SAT area, VAT area, and AM area were found to be significantly associated with being overweight and/or obese (BMI ≥ 25). The findings highlight that PFF hold promise as a imaging biomarker to identify individuals at risk of T2DM and being overweight and/or obese. Monitoring PFF may assist clinicians in formulating more precise strategies for prevention and treatment. Additionally, in individuals without diabetes, focusing on SAT area, VAT area and AM area may help identify potential health risks and provide a basis for targeted weight management and prevention measures.

The human body's fat storage is primarily composed of SAT and VAT, with SAT accounting for the majority of human adipose tissue (31, 32). Aside from the main subcutaneous and visceral fat depots, *de novo* adipogenesis will also occur in other parts (31). When the fat accumulation exceeds the expansion capacity of the SAT, excess lipid can accumulate in ectopic fat depots such as bone, liver, pancreas, and skeletal muscle (33). These fat depots might not exist independently and are influenced by age, gender and BMI, etc. In our study, it was showed that after adjusting for age, sex and BMI, there were varying degrees of correlations among these fat depots, particularly, VAT was correlated with all the other quantitative parameters. These findings indicated that VAT may be a marker of ectopic fat deposition (8).

Previous studies have showed that the accumulation of VAT is an risk factor of T2DM, while the expansion of SAT may be a protective factor (34, 35). However, abnormal expansion of SAT may also be a part of the pathological process. The insufficient capacity of SAT to recruit and/or differentiate available precursor cells may lead to hypertrophic expansion of the cells, resulting in IR and an increased risk of T2DM (33). As expected, in this study, we observed higher levels of VAT in the T2DM group, and VAT area is an independent identifying factor of T2DM, with an AUC value of 0.685. These findings were consistent with previous studies (36–38).

Compared to SAT, VAT exhibits higher metabolic activity and plays an more important role in regulating whole-body metabolism (39). The accumulation of VAT increases the risk of T2DM, which may be related to the following mechanisms. The venous blood of VAT drains into the liver through the portal vein, supplying the liver with free fatty acids and adipokines secreted by VAT cells. As a result, VAT accumulation can expose the liver to high concentrations of free fatty acids and glycerol, which will lead to reduced uptake of insulin by the liver (aggravating hyperinsulinemia), increased triglyceride-rich lipoproteins, and excessive stimulation of hepatic gluconeogenesis, ultimately increasing the risk of T2DM and hyperglycemia (8, 39, 40). In addition, VAT accumulation is accompanied by more inflammatory cell infiltration, which leads to an imbalance in the expression of pro-inflammatory and anti-inflammatory adipokines, thus interfering with glucose metabolism and increasing the risk of T2DM (8, 39, 41). Therefore, controlling and reducing the accumulation of VAT can reduce the risk of T2DM by improving insulin sensitivity, reducing the level of inflammation and reducing the release of fatty acids.

In the present study, we observed that the SAT area is not associated with T2DM, which is consistent with previous findings (9). Additionally, we observed that SAT FF in T2DM patients was lower than in non-T2DM patients. However, the difference in SAT FF between the two groups was no longer significant after adjusting for age and sex. This suggests that factors such as age and gender may play a certain role in interfering with this association.

Hepatic fat deposition is characterized by the accumulation of TG within hepatocytes (42). Previous studies have shown an association between hepatic fat deposition and T2DM. In this study, we found that patients with T2DM had higher HFF compared to non-T2DM patients. Sarma et al. (43) revealed that T2DM is related to the increase of fat deposition in liver, pancreas and viscera, and may be a contributing factor to IR in T2DM. Levelt et al. (7) found that diabetes, regardless of obesity, is associated with an increase in hepatic triglyceride content. Cao et al. (26) observed that patients with T2DM and prediabetes have higher HFF compared to individuals with normal glucose tolerance. Our findings were consistent with these results. However, it was found that HFF was not the independent risk factor for T2DM, which is consistent with the findings of Zheng et al. (9). We speculate that hepatic fat deposition may not be an independent mechanism in the pathogenesis of T2DM, and its role may be influenced by VAT, which collectively play important modulatory roles in T2DM development. Previous studies have indicated that the metabolites of VAT are mainly metabolized through portal vein circulation. Excessive accumulation of VAT will lead to the liver being exposed to high concentrations of free fatty acids and glycerol. When hepatic lipid supply exceeds the rate of lipid oxidation and output, the accumulate of TG in the liver as lipid droplets, resulting in the development of fatty liver (8, 39, 40, 44). Therefore, the relationship between HFF and T2DM may be mediated by VAT. However, we also recognized that there is significant heterogeneity in the pathogenesis of hepatic fat accumulation, leading to varying relationships between fatty liver and glucose metabolism. Previous studies has indicated that higher liver fat content may not increase the risk of IR and diabetes in certain patients with a genetic predisposition to hepatic steatosis. Conversely, severe IR and high risk of T2DM have been observed in patients with hepatic steatosis caused by an unhealthy lifestyle and excessive accumulation of VAT (45). Furthermore, Stefan and colleagues have identified that the characteristic of IR associated with metabolically unhealthy fatty liver is elevated levels of fetuin-A, and this phenotype may differ from that of IR associated with visceral obesity, which is primarily characterized by low plasma adiponectin levels (46). Therefore, adopting new risk stratification approaches to distinguish between hepatic fat deposition and visceral obesity may contribute to a better understanding of the relationship between hepatic fat accumulation and T2DM, and provide more targeted prevention and treatment strategies.

Compared to the liver, the pancreas appears to be more susceptible to fat accumulation (47). Increasing evidence suggests that pancreatic fat deposition may be associated with lipotoxicity, IR and inflammation, which could contribute to the development of glucose metabolism disorders (48). So far, the evidence about the relationship between pancreatic fat accumulation and T2DM is not consistent. Some cross-sectional studies based on CT and MRI have indicated that compared with non-T2DM patients, T2DM patients have higher PFF (9, 10, 12, 43, 49). Our research has reached the similar conclusion. In addition, Yi et al. (50) indicated that T2DM patients with longer disease duration have higher levels of pancreatic fat accumulation compared to those with shorter duration. However, a recent MRI study, which based on age, gender, and BMI matched T2DM patients (131 cases) and non-T2DM patients (135 cases), did not observe the difference of PFF between the two groups by placing ROIs in the head, body, and tail of the pancreas on MRI FF mapping (14). This finding contradicts our research results. This discrepancy may be attributed to variations in the methods used to assess pancreatic fat deposition, uneven distribution of pancreatic fat deposition, as well as differences in race, gender, and genetic factors. It is gratifying to note that in this study, we discovered an independent association between PFF and T2DM, with PFF demonstrating the best performance in identifying T2DM. This suggests that an increase in PFF may more accurately reflect the deterioration of adipose tissue quality, and thus indicating the raising risk of T2DM development. These findings underscore the critical role of PFF in the pathophysiology of T2DM and offer new insights for the prevention and treatment of T2DM. The fat content of pancreatic endocrine cells is considered a key factor in the pathogenesis of T2DM (51). Previous studies have indicated that elevated levels of triglycerides have lipotoxic effects on islet β-cells, leading to impaired endocrine function and reduced insulin secretion (52). Furthermore, exposure of the pancreatic islets to high levels of fatty acids may result in β-cell dedifferentiation, which is also considered a potential mechanism for T2DM (51). Ectopic fat accumulation within endocrine and exocrine organs occurs after the obesity-associated exhaustion of the adipogenic capacity of adipocyte precursors within bona fide fat depots (53). The paracrine action of lipids within adipocytes and acinar cells may contribute to local inflammation and impairment of β-cell function through the release of adipokines and other metabolite (51, 54, 55). Therefore, controlling or reducing pancreatic fat content may contribute to better glycemic control and improved metabolic health.

BMAT, a metabolically active and insulin-sensitive unique fat depot, may play a role in whole-body energy metabolism and glucose homeostasis (56, 57). Similar to other fat depots, marrow adipocytes release various adipokines (such as leptin, adiponectin, etc.) and free fatty acids through endocrine and paracrine pathways, regulating insulin sensitivity and mediating IR (58, 59). In addition, pro-inflammatory cytokines released by marrow adipocytes might mediate systemic chronic inflammation, which is considered a pivotal factor in the progression of T2DM and its complications (60, 61). In our study, we observed significant differences in BMAT content between patients with and without T2DM. This finding aligns with previous research (30, 62, 63), indicating higher levels of BMAT in patients with T2DM. Yet, the study by de Araújo et al. (18) showed different results, and they observed that there was no difference in BMAT content at the L3 vertebra between T2DM (28 cases) and control groups (24 cases) by magnetic resonance spectroscopy. Possible reasons for this disparity could include differences in measurement methods and locations, as well as their limited sample size. However, after adjusting for age and gender, the difference in BMAT FF between the T2DM group and non-T2DM group was no longer significant. We speculate that this may be due to the older age of the patients in the T2DM group and the possibility that bone marrow may not be the primary site of fat accumulation in T2DM.

As the main effector organs of insulin, skeletal muscle plays an important role in maintaining local and overall glucose homeostasis and IR. The existing research on the relationship between body composition and T2DM has primarily focused on adipose tissue, with limited understanding of the independent role of skeletal muscle in predicting or diagnosing T2DM. Our study demonstrated that the both AM FF and AM area were higher in T2DM patients compared to non-T2DM patients. This results is consistent with previous research findings (15, 64). Currently, the underlying mechanisms of skeletal muscle in IR and the development of T2DM remain unclear. Previous studies have suggested that IR in skeletal muscle may manifest prior to β-cell failure and elevated blood glucose in T2DM (65). IR in liver and muscle can lead to increased lipolysis and release of free fatty acids, as well as hyperglycemia. This process further stimulates ectopic fat deposition in the liver and muscles. To cope with the IR of the periphery and liver, the pancreas secretes more insulin, which leads to hyperinsulinemia. This process stimulates ectopic fat deposition in the liver and muscles again, forming a vicious circle (66, 67). In skeletal muscle and liver, the increase of fat storage may be related to the increase of IR, which results in the inhibition of glucose uptake in muscle cells, the increase of hepatic gluconeogenesis and the decrease of glycogen synthesis (66, 68). In addition, cytokines and adipokines released by adipose tissue can also regulate insulin sensitivity in liver and skeletal muscle (66). Given the significant differences in age and gender distribution between the two groups, which may have an impact on the experimental results, we performed age and gender adjustments. After adjustment, we found that the differences in the AM area and FF between the two groups were no longer significant. To further investigate the predictive value of different fat depots for T2DM in different age and gender groups, it is necessary to expand the sample size and conduct subgroup analysis stratified by age and gender.

To explore the associations between seemingly healthy individuals and multiple body compositions, we further divided the patients in the non-T2DM group into two subgroups based on BMI. We observed that a close correlation between higher SAT, VAT, and AM areas and overweight and/or obesity. Therefore, focusing on the SAT area, VAT area, and AM area in non-diabetic patients may help identify potential health risks and provide a foundation for targeted weight management and preventive measures.

## 5 Strengths and limitations

The strength of our study is that we used MRI FF mapping to non-invasively and accurately assess body composition, including AM, BMAT, and ectopic fat deposits, and to explore the relationship between multiple body composition factors and T2DM. To avoid the influence of uneven distribution of hepatic fat and pancreatic fat contents, we employed a 3D semi-automatic segmentation method based on MRI FF mapping to quantify whole hepatic fat and whole pancreatic fat. Furthermore, DeLong test was employed to compare the differences of the AUC values of VAT area, and PFF, evaluating their diagnostic performance in T2DM. Our results emphasize the critical role of PFF in the onset and progression of T2DM, and hold promise as a potential imaging biomarker for the prevention and treatment of T2DM. However, there were several limitations to our study. First of all, our study is based on a cross-sectional design, which cannot establish causality. Therefore, further longitudinal studies are needed to validate the findings of our research. Secondly, subjects in our study were from a single central hospital in China, and larger scale studies are needed to verify whether our results can be extrapolated to other ethnic populations. Thirdly, the distribution of fat in T2DM patients differs by gender, thus it is necessary to further expand the sample size and subgroup analysis stratified by age and gender to explore the predictive value of different adipose depots for T2DM in different ages and sexes. Finally, it is not feasible to evaluate the impact of drug treatment on body composition parameters due to the small sample size of T2DM patients undergoing treatment and the absence of a comparison of these parameters before and after treatment. Therefore, further large-scale and prospective studies are necessary to comprehensively and thoroughly investigate the influence of diabetes treatment on the body composition of T2DM patients.

## 6 Conclusions

In this study, we investigated the associations of ectopic fat deposition, AM, and BMAT with the incidence of T2DM. Our results showed that VAT area and PFF were independent risk factors for prediction of T2DM, and PFF showed the best diagnostic performance. Therefore, PFF based on MRI FF mapping could be a potential radiological biomarker to help clinicians to more accurately screen high-risk T2DM individuals, provide more personalized treatment, and monitor the therapeutic effect. However, in order to better apply PFF to clinical practice, further prospective studies are needed to investigate the role of PFF in the pathological mechanism of T2DM. Additionally, in individuals without diabetes, focusing on SAT area, VAT area and AM area may help identify potential health risks and provide a basis for targeted weight management and prevention measures.

## Data availability statement

The raw data supporting the conclusions of this article will be made available by the authors, without undue reservation.

## Ethics statement

The studies involving humans were approved by the Ethics Committee of the First Affiliated Hospital of Dalian Medical University. The studies were conducted in accordance with the local legislation and institutional requirements. The Ethics Committee/Institutional Review Board waived the requirement of written informed consent for participation from the participants or the participants' legal guardians/next of kin because this study is a retrospective study and informed consent is exempted.

## Author contributions

QA: Conceptualization, Data curation, Formal analysis, Investigation, Methodology, Project administration, Supervision, Validation, Writing—original draft, Writing—review & editing. Q-HZ: Conceptualization, Methodology, Writing—original draft, Writing—review & editing. YW: Investigation, Project administration, Writing—original draft. H-YZ: Investigation, Writing—original draft. Y-HL: Investigation, Writing—original draft. Z-TZ: Investigation, Writing—original draft. M-LZ: Investigation, Writing—original draft. L-JL: Writing—review & editing. HH: Writing—review & editing. Y-FY: Writing—review & editing. PS: Writing—review & editing. Z-YZ: Writing—review & editing. Q-WS: Writing—review & editing. A-LL: Conceptualization, Writing—review & editing.
